# Book Reviews

**Published:** 1903-07

**Authors:** 


### BOOK REVIEWS.

PROGRESSIVE MEDICINE. June, 1903. Lea Bros. & Co., New York and Philadelphia.

The contributors to this volume of the Progressive Medicine series include John G. Clarke, late of Johns Hopkins but now of Philadelphia; W. B. Coley, Alfred Stengel, Edward Jackson. The subjects considered are Surgery of the Abdomen, including Hernia, by Coley; Gynecology, by Clarke; Diseases of the Blood and Ductless Glands; the Hemorrhagic Diseases, Metabolic Diseases, by Stengel; Ophthalmology, by Edward Jackson.

The latest information on these important subjects has been collected, sifted and systematically set forth. This series can be commended to the progressive physician as offering a storehouse of current medical usage and opinion.

### STATE EXAMINING BOARD FOR TRAINED NURSES.

A bill has just passed both branches of the Virginia Legislature providing for the creation of an Examining Board for Trained Nurses, to be composed of five members, to be appointed by the Governor from twelve nominations submitted to him by the Virginia State Association of Graduate Nurses. Any nurse who graduates prior to January 1, 1904, is exempted by a provision of the bill from examination. It now goes to the State's Chief Executive for his signature. One section of the proposed new law reads: "This act shall not be construed to affect, or apply to, the gratuitous nursing of the sick by friends or members of the family: and also it shall not apply to any person nursing the sick for hire, but who does not in any way assume to be a registered or graduate nurse."

